# Pseudo-enantiomeric chiral components and formation of the helical micro- and nanostructures in charge-transfer complexes

**DOI:** 10.1098/rsos.171499

**Published:** 2018-03-14

**Authors:** Jerzy J. Langer, Grzegorz Hreczycho

**Affiliations:** 1 Faculty of Chemistry, Laboratory for Materials Physicochemistry and Nanotechnology, Adam Mickiewicz University in Poznań, Umultowska 89b, Poznań, 61-614, Poland; 2 Faculty of Chemistry, Laboratory for Chemistry and Technology of Inorganic Polymers, Adam Mickiewicz University in Poznań, Umultowska 89b, Poznań, 61-614, Poland

**Keywords:** conductive CT complexes, helical micro- and nanostructures, TCNQ

## Abstract

Helical organic micro- and nanostructures are formed by a charge-transfer complex, cinchonidine-TCNQ. These unusual forms result from the chirality, the steric structure and specific interactions of cinchonidine molecules. These materials are semiconductors (10^−4^ S cm^−1^), with the typical absorption spectra in IR and UV-vis, but also have a characteristic of CD spectrum. Surprisingly, conductive micro and nano helices are not formed in pseudo-enantiomeric cinchonine, i.e. the complex of cinchonine and TCNQ.

## 1. Introduction

Organic semiconductors: tetracyanoquinodimethane (TCNQ) charge-transfer (CT) complexes are, for a long time, in the focus of interest of our laboratory, owing to their unique electrical and optical properties, including high anisotropy resembling low-dimensional systems (quasi-one-dimensional). Normally, complexes with chiral donors crystallize as needles [1–4].

We present here unique helical micro- and nanocrystals, which are formed as a complex of cinchonidine (CND, a chiral electron donor) and tetracyanoquinodimethane (TCNQ, as an electron acceptor). TCNQ radical-ion salts (RIS) and charge transfer complexes are well known as highly conducting organic materials (mainly semiconductors) [2,5,6] with the electrical conductivity reaching a value up to 10^3^ S cm^−1^. These low-molecular weight materials are soluble in organic solvents and easily processable.

The helical symmetry is normally not observed in the case of monocrystals without defects.

Surprisingly, similar helical microcrystals of TTF-TCNQ complex can also be obtained without any chiral components, with a two-phase method (TTF dissolved in hexane and TCNQ in acetonitrile) [7]. In this case, no asymmetric additives and no asymmetric physical interactions (magnetic or electric field) are applied, except for stirring, which generates swirls (vortices) with the same direction of rotation (helicity), influencing the nucleation, growth and assembly of nanoparticles, because of the conservation of total angular momentum and Coriolis effect. That is why the helicity of all microcrystals is the same, as reported.

Helicity is also appearing as an element of symmetry of a superlattice. Helical molecular ordering is attributed to the cholesteric liquid crystals [8–10] and this type of materials has been applied by Shirakawa and co-workers to obtain helical polyacetylene, which was synthesized in a chiral environment with cholesteric liquid crystal phase used as the asymmetric reaction field. Following this procedure, a helical form of polyacetylene has been prepared in a template synthesis with chiral nematic liquid crystals [11].

Helical supramolecular structures formed owing to a self-assembly process were observed in fatty acids [12], N-alkylaldonamide fibre gels [13] and charged poly(styrene)-poly(isocyanodipeptide) block copolymers [14], where chiral macromolecules form helical superstructures.

## 2. Approach

7,7,8,8-tetracyanoquinodimethane (TCNQ), cinchonidine (CND) and cinchonine (CN) were purchased from Sigma–Aldrich. Before use, TNCQ, CND and CN were re-crystallized according to the standard procedures. Other chemical reagents of analytical grade, purchased from Sigma–Aldrich were used directly as received.

The complex of a helical morphology (CND-TCNQ) was prepared with an excess of the acceptor component (TCNQ) reaching the molecular ratio CND/TCNQ between 1 : 2 and 1 : 4. The product was crystallized with acetonitrile under the argon shielding, lowering slowly the temperature within at least 12 h. If necessary, a partial, controlled evaporation of solvent was also applied.

Elemental analysis data:
Found: 73.02% C, 4.72% H, 19.12% N.
Calculated for CND-TCNQ (2 : 3): 73.98% C, 4.70% H, 18.65% N, with the best fit for the structure CND-TCNQ-CH_3_CN of a stoichiometry 2 : 3 : 1, 73.47% C, 4.78% H, 19.16% N.
UV-vis (nm): 394, 681, 743, 761, 842.
FTIR (selected; KBr, cm^−1^): 2983, 2881 (C-H; CND), 2270 (C=N; CH3CN), 2237, 2220, 2186 (C=N; TCNQ).
EPR: single symmetric line (asymmetry 0.944), *g* = 2.0034, Δ*H* = 0.24 mT.
By the same way, CN-TCNQ complex was prepared.
Elemental analysis data:
Found: 72.04% C, 3.31% H, 23.03% N.
Calculated for CN-TCNQ (1:4): 72.42% C, 3.44% H, 22.69% N, with the best fit for the ratio CN/TCNQ 1:4.5: 72.26% C, 3.32% H, 23.09% N.
UV-vis (nm): 392, 478, 680, 743, 761, 842.
FTIR (selected; KBr, cm^−1^): 2930 (C-H; CN), 2225, 2200, 2185, 2170 (C=N; TCNQ).
EPR: single symmetric line (asymmetry 1.019), *g* = 2.0027, Δ*H* = 0.49 mT.
IR spectra were recorded on FTIR Bruker IFS 113v spectrometer (a solid sample suspended in a KBr disc).
The UV-vis spectra (10^−4 ^mol dm^−3^ in acetonitrile) were recorded on Shimadzu N160.
The CD spectra were measured with the aid of Dichrograph Mark II, Jobin-Yvon.

Electron microscope examinations were performed using a Jeol JMS-50A scanning microscope operated at SEI mode with a voltage of 20 kV and a current of about 10 pA. The sample was spread onto the surface of a holder and observed directly with no further treatment (carbon or gold films were not deposited). The electrical conductivity of the complex examined is high enough to get good quality SEM images. However, to stabilize nanocrystals a gold film was evaporated.

High energy electron diffraction (HEED) experiments were performed with the aid of a Jeol JEM-7A electron microscope operated at 80 kV (and also at 120 kV).

The diffraction pattern depends strongly on how the crystal is oriented against the electron beam. The layer lines, nearly parallel to the helix axis, are only observed when the electron beam is almost parallel to the axis **a** (figure 3*b*). A cross-like pattern, which is typical for the helical ordering, appears when the electrons pass along the axis **b**. Unfortunately, this is the longest distance (about 5000 nm) for electrons to penetrate it. Thus, the pattern observed is the result of a perturbation of the incident electron beam owing to surface reflection, not to diffraction. The pattern is continuous and not discrete, and no spots are observed. The picture is better when the electron beam is exactly parallel to the axis **a** and locally perpendicular to the surface; however, it suffers from lack of details in the central part of a diffraction pattern. Now electrons have to pass the shortest distance (tiny nanocrystals are fully transparent), but still, we are not able to get a good quality diffraction pattern. This is caused by the instability of the structure examined.

## 3. Results and discussion

### 3.1. Unique helical morphology

**Figure 1.**
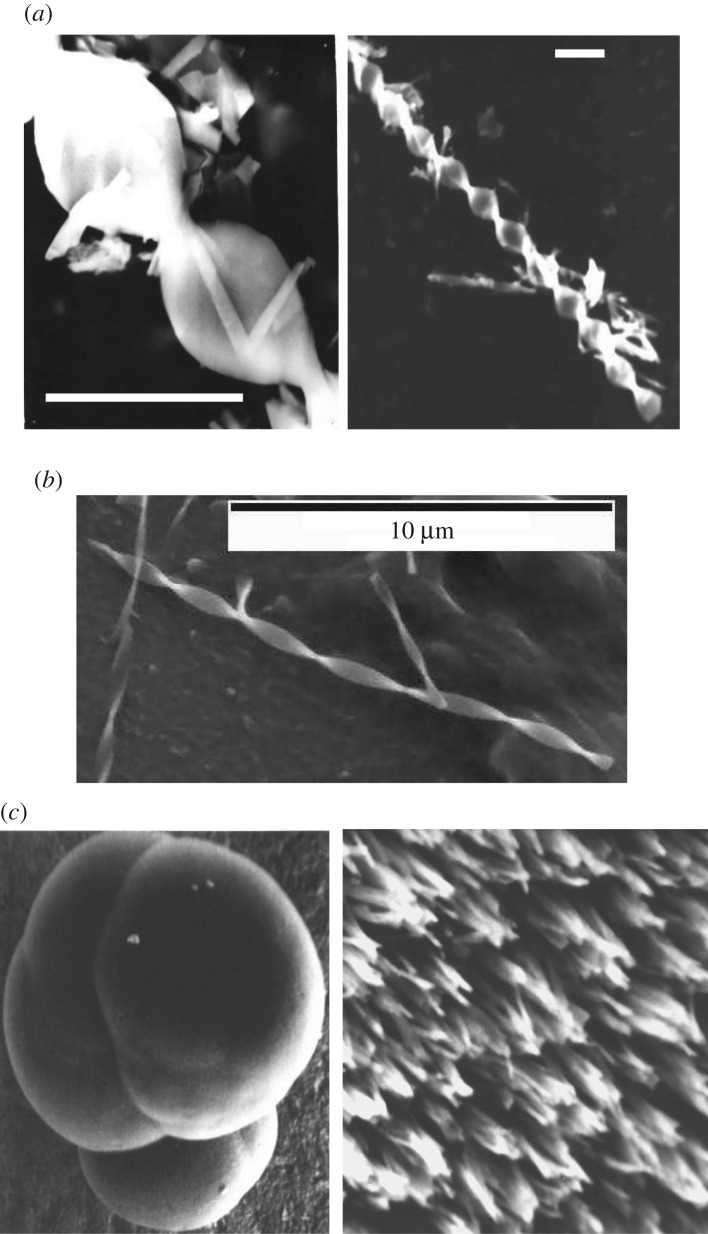
Helical structures of a complex CND-TCNQ (SEM micrographs; scale bar corresponds to 10 µm): micro helices of a periodicity of 20 µm (*a*) and 5 µm (*b*), micro helical structures at the surface of a spherulite (*c*).

Helical microcrystals are formed in cinchonidine-TCNQ CT complex not only as single helices of maximum dimensions 300–600 µm × 5 µm × 1 µm but also, in some circumstances, as spherulites (figure 1). The periodicity of a helix is almost constant for all the crystals in a specimen and amounts to about 20 µm for the biggest crystals and about 5 µm or less for crystals of a nanometre thickness (dimensions: 10–20 µm × 0.4–2 µm ×  0.05–0.35 µm), obtained in separate experiments. In most cases, the ratio of the period of a helix to its diameter is constant and amounts to about 10. However, the values between 2 and 4 can also be observed, particularly for the biggest crystals.

The complex of helical morphology is prepared according to a general standard procedure [3,4], with no special treatment (as described above). The helices are totally self-assembling structures.

Surprisingly, the analogous CT complex of pseudo-enantiomeric cinchonine and TCNQ does not form helices.

### 3.2. The molecular structure and properties

The stoichiometry of the helical complex CND-TCNQ is 2 : 3, as we can conclude from the elemental analysis, which corresponds to the formula 2CND-3TCNQ*CH_3_CN. It crystallizes in the form of micro and nano helices. Measurements of the I-V characteristics and the electrical conductivity (*σ*) for isolated single microcrystals along the helix axis have been performed using the two-electrode method (because of small dimensions of the crystals examined) with a set-up equipped in an optical microscope and a micromanipulator. We found the electrical conductivity to be relatively high, i.e. *σ* = 2.5*10^−4^ S cm^−1^ (at 300 K), and sensitive to temperature with the activation energy *E*_a_ of 0.2 eV in the range of 264–300 K. Below 264 K, the activation energy *E*_a_ is only 0.02 eV. This is a characteristic feature of the electrically conducting TCNQ complexes with specific stoichiometry and ordering the electron-donor and electron-acceptor components. The group includes complexes with quinoline derivatives [2,6], which are related to CND-TCNQ and CN-TCNQ. A characteristic feature of this group is the ordering of the molecules of the acceptor (here TCNQ), in the limited (or absence of) order in the system of donor molecules (CND). Usually, the acceptor forms columns of dimers with one excess electron (TCNQ^0^-TCNQ^−^) [5,6]. Such a structure has been found in many TCNQ complexes (non-helical) of the same type and of a comparable electrical conductivity [2,5,15].

This is adequate for UV-vis, IR and EPR spectra, but also the electrical conductivity of CND-TCNQ and CN-TCNQ: absorption at 842 nm, 761 nm, 743 nm and 681 nm assigned to TCNQ^−^ formed owing to charge-transfer CT; a multiple IR absorption at 2225–2237 cm^−1^ assigned to TCNQ^0^ ([15]: 2225 cm^−1^), approximately 2185 cm^−1^ assigned to TCNQ^−^ ([15]: 2187 cm^−1^); single symmetric EPR line CND-TCNQ *g* = 2.0034 ([15]: 2.0034–2.0040), Δ*H* = 0.24 mT; CN-TCNQ *g* = 2.0027, Δ*H* = 0.49 mT.

Optical and electrical properties of a complex of pseudo-enantiomeric cinchonine (CN) and TCNQ are similar, except the helical structure.

### 3.3. Intermolecular interactions

**Figure 2.**
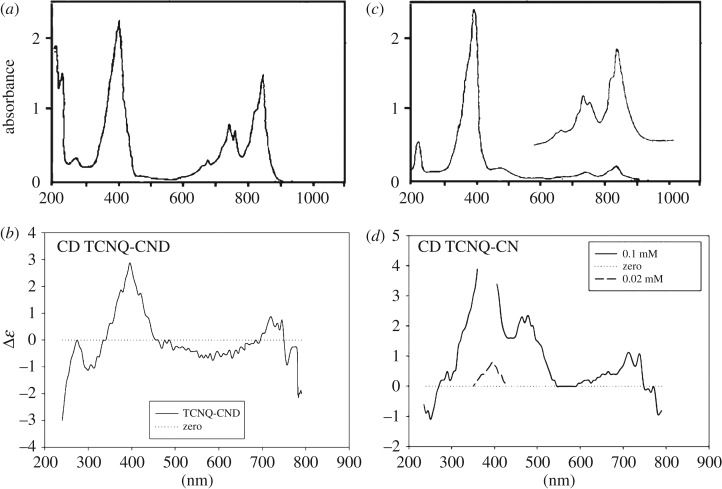
UV-vis and CD spectra of CND-TCNQ in acetonitrile (0.1 mM), (*a*) and (*b*), respectively; UV-vis and CD spectra of CN-TCNQ in acetonitrile (0.1 mM), (*c*) and (*d*), respectively.

Strong interactions between TCNQ molecules and quinoline residues are observed in both complexes in solutions and solid samples. However, here we focus our attention mainly on helical forms of CND-TCNQ. The infrared (IR) spectrum consists of broad and intensive absorption peaks near 1100, 1320 and 1550 cm^−1^ assigned to electron–phonon interactions. The absorption bands in the UV-vis region, corresponding to the CT and LE transitions, where CT are excitations in the charge transfer complex, and LE are localized excitations of individual molecules, are observed at 600–900 nm, below 500 nm and about 400 nm, respectively, [5] with maxima at 847 nm (11 806 cm^−1^), 761 nm (13 141 cm^−1^), 743 nm (13 459 cm^−1^) and 680 nm (14 706 cm^−1^) for CT and 394 nm (25 381 cm^−1^) for LE (figure 2*a*). CT corresponds to excitations of TCNQ · radical ions in the complex formed owing to the charge transfer between CND or CN and TCNQ, while LE are intramolecular excitations of individual TCNQ molecules—here, the most intensive absorption at 394 nm (CND-TCNQ) or 392 nm (CN-TCNQ) assigned mainly to excitation of neutral molecules TCNQ^0^ [5,16].

**Figure 3.**
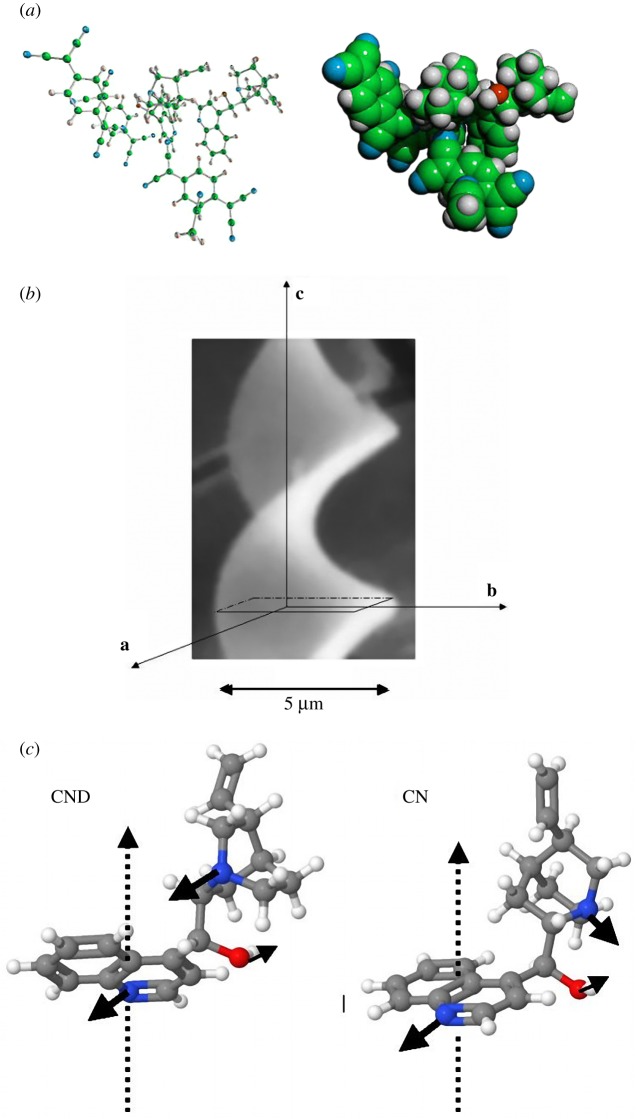
A computer model of the complex CND-TCNQ (2CND-3TCNQ*CH_3_CN) with the optimized geometry (*a*); CND-TCNQ micro helix—**a**, **b**, **c** directions (*b*); the direction π–π interactions of quinoline moieties and hydrogen bonding in CND and CN molecules—models (*c*). The computer modelling has been performed with the ChemSite program.

**Figure 4.**
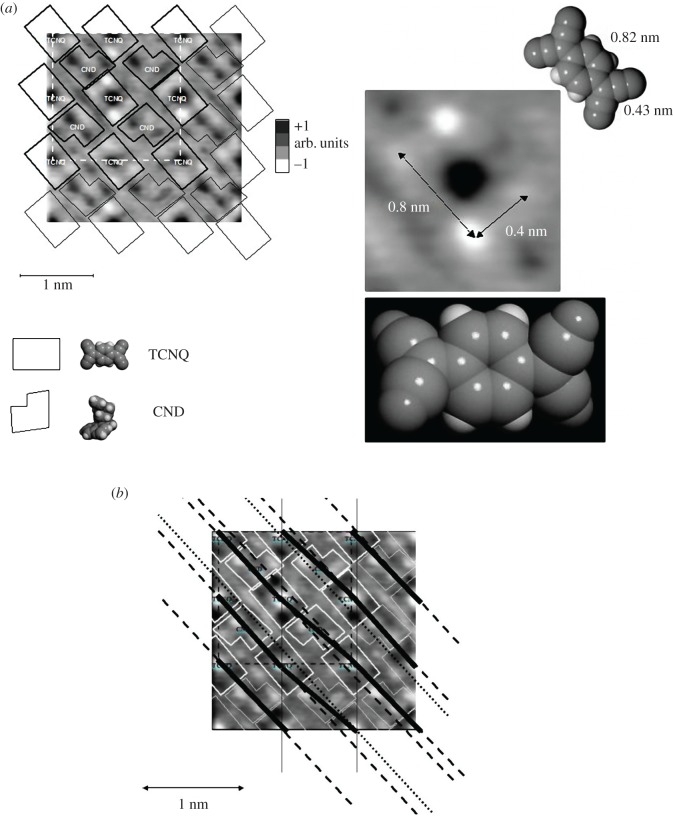
A two-dimensional potential map (a cross-section at *c* = 0 found owing to Fourier analysis of HEED data) for the helical complex examined: possible positions of CND and TCNQ molecules are identified (*a*). More detailed analysis discloses perturbation of the structure, owing to helical morphology (*b*).

All the absorption bands are optically active and can also be observed in the CD spectrum, figure 2*b*. CND-TCNQ complex shows negative and positive Cotton effects for π-π* and n-π* excitations in the range of 250–500 nm, as well as for CT transitions at the wavelength of 600–800 nm which cannot be attributed to cinchonidine molecules (the Cotton effect of pure CND is quite different and it does not appear above 420 nm). One can conclude that the interactions between CND and TCNQ are strong enough, even in the solution at a very low concentration (10^−4^ mol dm^−3^ and below). Close contact interactions between TCNQ and aromatic quinoline moiety of CND are energetically preferred to that of quinuclidine moiety, according to the literature data [15] and the results of a computer model (figure 3). This stabilizes the structure of the complex in solution, at least in the time scale of UV-vis experiments. In the solid materials, the interactions result in stacking of TCNQ^0^ and TCNQ^−^ molecules (usually as dimers in this type of complex [6,15]) surrounding quinoline residues of CND, oriented approximately along the axis, which is close to the main crystal growth direction (figure 4). The conclusion is consistent with the electrical conductivity measurements, but also with a high optical anisotropy and polarization effects, observed in single isolated CND-TCNQ helical structures with the aid of a polarizing microscope.

### 3.4. Surprising cinchonine-TCNQ complex

It is interesting that TCNQ and cinchonine (CN), an alkaloid related to CND (pseudo-enantiomer), do not form a complex of the same properties like CND-TCNQ, but a different stoichiometry CN/TCNQ 1 : 4 and morphology. The charge transfer interactions are not so strong in this case, what is concluded from the UV-vis spectrum of the CN-TCNQ complex (10^−4 ^mol dm^−3^ in acetonitrile, figure 2*c*): the absorption at 842 nm (11 877 cm^−1^), but also at 761 nm (13 141 cm^−1^), 743 nm (13 459 cm^−1^) and 680 nm (14 706 cm^−1^), assigned to the CT transitions, is much weaker than in CND-TCNQ complex. A weak absorption at 478 nm (20 921 cm^−1^) is related to the formation of α,α-dicyano-p-toluoyl cyanide anion, the product of decomposition of complexes with TCNQ anion radicals (not observed in CND-TCNQ). The most intensive LE absorption is observed at 392 nm (25 510 cm^−1^), which corresponds mainly to the excitation of neutral molecules in TCNQ^0^. Its maximum is a bit blue-shifted in comparison to that of CND-TCNQ, because of weaker interactions with CN molecules. However, the interactions are still strong enough to generate the Cotton effect (figure 2*d*), which is different in the sign and the amplitude than those for CND-TCNQ. The difference between cinchonine and cinchonidine complexes is also observed in the intensity of the EPR signal (registered in both cases as a single line of comparable spectral parameters: *g* = 2.0027, Δ*H* = 0.49 mT), which is much stronger for CND-TCNQ. Strong charge transfer interactions and a difference in hydrogen bonding (figure 3*c*), together with the steric effect (molecular compatibility), seem to play a crucial role in the formation of the helical CT complex with cinchonidine (CND-TCNQ), and not pseudo-enantiomeric cinchonine (CN-TCNQ).

### 3.5. Electron diffraction studies

The high energy electron diffraction experiments (HEED) were performed on isolated CND-TCNQ micro helices. Electrons, negatively charged particles, interact with matter through the Coulomb forces. When the electrons pass through the sample, they are scattered by the internal electrostatic potential, both on the positively charged atomic nuclei and the surrounding electrons (located at atomic and molecular orbitals). The same is possible on a gradient of molecular electric field, generated by the charge-transfer structure, here owing to the interaction of the electron acceptor (TCNQ) and the electron donor (CND). As in many other organic compounds, including low-dimensional organic conductors [17,18], the ordered structure of CND-TCNQ is highly sensitive to the impact of electrons and we are unable to get good quality HEED patterns. The damage of a helix is not caused by a thermal effect. No change in morphology is observed by heating the sample directly to the melting point (decomposition) at 445 K—the helical structure remains unchanged. However, there is a high sensitivity of the spiral in relation to the current flow. We observed a similar shape disruption in single-helix experiments during electrical measurements as in the TEM chamber. The maximum current density (measured directly) through a single CND-TCNQ micro helix does not exceed 5 A cm^−2^.

Thus, due to instability, the results of HEED are of low quality, leading to limited data. One can find the distance between TCNQ molecules in the stack of about 0.35 nm, similarly the thickness of TCNQ-cinchonidine bimolecular system, which is nearly 1 nm. This is close to the results of direct measurements with STM for other conducting TCNQ complexes, e.g. TTF-TCNQ [19]. Despite technical problems, analysing the diffraction pattern more closely (using Fourier transform), one can get the low-resolution two-dimensional map (a two-dimensional cross-section) of electron density distribution (the diffraction of electrons is due to different electrical potential), which corresponds roughly to the ordering of TCNQ and CND molecules in the complex. One can identify individual molecules, particularly such specific as TCNQ. Surprisingly, these molecules are distorted and not planar in the crystal lattice/superlattice (figure 4*a*). We also note an opposite orientation of CND molecules in neighbouring layers.

The molecular ordering in micro helices is undoubtedly observed with a polarized light as an optical anisotropy, typical for helical structures. The sample examined with a polarizing optical microscope shows the effect of optical activity only below the melting point (445–446 K). The pattern observed results from a helical structure and it disappears after melting. The effect is irreversible when cooling the sample. The most probable conclusion is that the complex CND-TCNQ forms a helical superlattice of the periodicity, which is equal to about 5 µm or even 20 µm, the same as observed for morphological forms. These values are too large to be detected with HEED experiments—much bigger than the area analysed which is usually a few square nanometres.

The helices are transparent, optically active and conductive. These make them potentially useful for optoelectronics, and other techniques where the light and the electric field are interacting, including nantennas [20–24] and metamaterials [25–28]. A system of small spherulites composed of densely packed helices (figure 1*c*) is potentially even more effective and promising.

A nantenna is designed to absorb specific radiation of the wavelength that is comparable to the size of the device, e.g. Idaho National Laboratories has designed a nantenna to absorb wavelengths in the range of 3–15 µm [20] (photon energies of 0.08–0.4 eV). A nantenna can absorb any wavelength of light efficiently provided that the size of the nantenna is optimized for that wavelength, e.g. nantennas can be used to absorb light at wavelengths between 0.4 and 1.6 µm which make up about 85% of the solar radiation spectrum [21].

## 4. Conclusion

The CT complex of cinchonidine and TCNQ (CND-TCNQ) is a helical organic semiconductor of low molecular weight. Surprisingly, the complex of cinchonine (which is a pseudo-enantiomer) and TCNQ (CN-TCNQ) does not form helices. This is because of the difference in orientation of hydrogen bonding versus direction of π-interactions of quinoline moieties in CND and CN molecules, which leads to helical morphology in CND-TCNQ and not CN-TCNQ.

Such an organic material of helical structure, the relatively high electrical conductivity, the optical chirality and strong electron–phonon interactions, are expected to be important for future materials (metamaterials), the electronics and optoelectronics, including nanoelectronic devices. Possible applications include optical control of the current flow, induction-active micro- and nanoelements, nano antennas (nantenna, a nanoscopic rectifying antenna to convert light to electric power) [20–24], ultra-miniature selective antennas for signal and power (e.g. heat) transfer, and also in the fabrication of chiral metamaterials [25–28].

## Data accessibility

Data are mostly included in the text. Part of it (HEED pattern) is available at (http://dx.doi.org/10.5061/dryad.4ts31) [29].
